# Characterisation of Microparticle Waste from Dental Resin-Based Composites

**DOI:** 10.3390/ma14164440

**Published:** 2021-08-08

**Authors:** Steven Mulligan, Jesús J. Ojeda, Gabriella Kakonyi, Steven F. Thornton, Keyvan Moharamzadeh, Nicolas Martin

**Affiliations:** 1 Academic Unit of Restorative Dentistry, School of Clinical Dentistry, Claremont Crescent, The University of Sheffield, Sheffield S10 2TA, UK; n.martin@sheffield.ac.uk; 2 Systems and Process Engineering Centre, College of Engineering, Swansea University, Swansea SA1 8EN, UK; j.j.ojedaledo@swansea.ac.uk; 3 Groundwater Protection and Restoration Group, Department of Civil and Structural Engineering, Sir Frederick Mappin Building, Mappin Street, The University of Sheffield, Sheffield S1 3JD, UK; g.kakonyi@sheffield.ac.uk (G.K.); s.f.thornton@sheffield.ac.uk (S.F.T.); 4 Hamdan Bin Mohammed College of Dental Medicine (HBMCDM), Mohammed Bin Rashid University of Medicine and Health Sciences (MBRU), Dubai P.O. Box 505055, United Arab Emirates; keyvan.moharamzadeh@mbru.ac.ae

**Keywords:** resin-based composite, pollution, microplastic, microparticle, particles size analysis, potentiometric titration, FTIR

## Abstract

Clinical applications of resin-based composite (RBC) generate environmental pollution in the form of microparticulate waste. Methods: SEM, particle size and specific surface area analysis, FT-IR and potentiometric titrations were used to characterise microparticles arising from grinding commercial and control RBCs as a function of time, at time of generation and after 12 months ageing in water. The RBCs were tested in two states: (i) direct-placement materials polymerised to simulate routine clinical use and (ii) pre-polymerised CAD/CAM ingots milled using CAD/CAM technology. Results: The maximum specific surface area of the direct-placement commercial RBC was seen after 360 s of agitation and was 1290 m^2^/kg compared with 1017 m^2^/kg for the control material. The median diameter of the direct-placement commercial RBC was 6.39 μm at 360 s agitation and 9.55 μm for the control material. FTIR analysis confirmed that microparticles were sufficiently unique to be identified after 12 months ageing and consistent alteration of the outermost surfaces of particles was observed. Protonation-deprotonation behaviour and the pH of zero proton charge (pH_zpc_) ≈ 5–6 indicated that the particles are negatively charged at neutral pH7. Conclusion: The large surface area of RBC microparticles allows elution of constituent monomers with potential environmental impacts. Characterisation of this waste is key to understanding potential mitigation strategies.

## 1. Introduction

Plastic microparticles, described commonly as microplastics, are recognised as key environmental pollutants, with concerns centred around negative impacts on aquatic life, soil, as transportation vectors for more toxic chemicals or pathogens and potential bioaccumulation in food webs [1,2,3,4]. Microplastic pollution was recognised over a decade ago and can be defined as the release of small (<5 mm) synthetic plastic particles [3]. Common sources of microplastic pollution in the environment include polymeric fibres released from the laundering of synthetic textiles [5], personal care products containing synthetic exfoliating microbeads and also the breakdown of macro-scale plastics already in the environment [6]. Microparticles containing plastics generated from the use of resin-based composite (RBC) should also be considered a pollution source requiring further investigation. Micro-scale and nano-scale particulates containing plastic polymers are generated in dentistry via clinical applications using dental materials containing plastic, namely, RBCs which enter the environment [7,8]. The mechanism of this pollution pathway is the small-scale but frequent release of plastic-based microparticles into municipal wastewater from dental treatments (removing old restorations or adjusting new restorations) and the subtractive fabrication (milling and grinding) of resin-based dental prostheses such as crowns, inlays and onlays. The extent and environmental impact of microparticles containing plastic that are generated from dentistry is poorly understood and an assessment of risk to the environment is not possible due to a lack of data.

RBC is made up of 60–80% inorganic glass filler particles coupled to and contained within an organic resin matrix. The resin matrix of RBC materials is methacrylate-based, composed of monomers of varying size, rigidity and hydrophilicity such as bisphenol A glycerolate dimethacrylate (BisGMA), urethane dimethacrylate (UDMA), hydroxyethyl methacrylate (HEMA) and triethylene glycol dimethacrylate (TEGDMA) [9,10]. Other components required for optimal polymerisation of the material, such as inhibitors and activators are also contained within the resin matrix (Table 1). The biocompatibility of resin-based dental materials, and more specifically the constituent monomers, has been discussed in the literature [11]. Synthesis of the monomer BisGMA, commonly used in RBC materials requires bisphenol A (BPA). BPA is associated with health-related problems when critical levels are reached due to its oestrogen-mimicking properties and the release of BPA from dental RBC and sealants has been reported [12,13,14,15].

RBC is used as a dental material that is directly placed and subsequently cured and set solid via radical vinyl polymerisation using a high-intensity visible blue light source (≥300 mw/cm^2^ and 480 nm) [16]. Through this process, the monomers reach an approximate level of 60–70% conversion, with the unreacted remainder trapped in the matrix as partly linked monomers [17,18,19]. RBC restorations can also be fabricated extra-orally in the form of an inlay, onlay or crown and subsequently adhesively bonded to the tooth. CAD/CAM (computer-aided design/computer-aided manufacturing) technology is used to mill or grind pre-polymerised blocks through a subtractive process that generates waste particulate powder [20,21].

RBC microparticulate waste is generated and released into the environment via two main pathways: (i) The clinical grinding of in situ RBC restorations with high-speed rotary abrasive burs/discs, either through the removal of failed/aged RBC dental restorations and/or the shaping, finishing and polishing of a directly placed restoration. (ii) The subtractive fabrication of prostheses (inlays, onlays, crowns, bridges and implant abutments) by milling and grinding pre-polymerised RBC ingots. It is estimated that in 2015, 800 million direct RBC restorations were placed; a figure based on industry sales figures [22]. It is recognised that ~6% of these restorations will fail within 10 years, equating to approximately 48 million restorations being removed or refurbished with resultant release of RBC particulates into municipal landfill, incineration and wastewater [23]. Assuming that the average weight of a RBC restoration is 0.3 g, a subsequent extrapolation would set the particulate waste generated and released into municipal wastewater to be up to 14.4 tonnes per year. Another meta-analysis study estimated that 32 million RBC posterior restorations placed in 2015 will require replacement or repairing within 10 years (2025), further highlighting the potential pollutant waste pathway of RBC microparticles [24].

Free or partly-linked monomers elute from the resin matrix of direct-placement restorations and by extension also from microparticulate waste [25]. One study that investigated the release of monomers from composite particulate waste estimated up to 360 μg/m^3^ of UDMA, 180 μg/m^3^ of Bis-GMA, 970 μg/m^3^ of TEGDMA and 1.28 μg/m^3^ of BPA were eluted into ethanol [26]. Elution is also increased by hydrolysis, photolysis and oxidation of the resin matrix [27] and accelerated by microbial biodegradation [28]. Microorganisms capable of facilitating biodegradation of plastic materials can readily be isolated from the environment [29,30] and would have the same impact upon dental microparticulate waste.

The recognised short- and long-term elution of monomers from RBC [31], the further elution caused by bacterial degradation mechanisms [32] and the large surface area of microparticulate waste are contributory to increasing the pollution potential of RBC waste particulates. It is well recognised that the constituent monomers of these RBC microparticulate have cytotoxic and genotoxic effects in high concentrations, however the full extent of this impact is difficult to ascertain [33,34,35]. To manage this pollutant route and prevent or mitigate any potential harm, it is important to better understand the nature of microparticulate waste from this source and the effect of its release into the environment.

The aims of this investigation were (i) to characterise the size and distribution of RBC microparticles that are created from simulated clinical use of conventional direct placement RBC and CAD/CAM RBC, (ii) to analyse the effect of ageing of the microparticles in water to simulate environmental release, and (iii) to assess the potential reactivity of the RBC microparticles in the environment and their potential to become toxic polluters and/or vectors for pollution.

## 2. Materials and Methods

RBC microparticles in solution were assessed at two points in time: comparing RBC released at the time of generation and following 12 months ageing in water. Ground particulate RBC waste was tested from two sources: (i) Direct-placement materials, ground using dental burs to simulate routine clinical use (Direct Placement), and (ii) Pre-polymerised RBC blanks, ground into a final shape using CAD/CAM technology (CAD/CAM). The RBCs in both states contained the same monomers and originated from two sources (Table 1):

1. In-house fabricated RBC (Control) specifically designed and fabricated with quantifiable constituents that were used for both the direct placement (Direct-Control) and the CAD/CAM samples (CAD/CAM-Control) (Table 2). Fabrication of the control composite followed an established protocol used by the research group in previous RBC experimental studies [36].
2. Commercially available direct and indirect RBC materials (COM). A direct placement RBC (Direct-COM) (Filtek Supreme XTE, dentine shade A3; 3M Oral Care, Irwindale, CA, USA) was chosen as this material as it contained all the monomers of the control RBC. CAD/CAM RBC (CAD/CAM-COM) (Lava Ultimate size 14L shade A3; 3M Oral Care, Irwindale, CA, USA) was also used to represent CAD/CAM RBC materials. It was not possible to obtain the full constituent data for the resin matrix of Lava Ultimate, although it is reported that this material contains UDMA as the principal monomer that is solely heat-polymerised and has 79 wt.% of zirconia-silica nanofillers [37]. Filtek Supreme XTE and Lava Ultimate RBCs were selected as being representative of their class in the market containing common constituents of current state-of-the-art RBC materials. The monomer component of Filtek Supreme XTE consists of BisGMA, UDMA, TEGDMA and ethoxylated BisGMA (BisEMA6). The fillers in Filtek Supreme XTE are a combination of non-agglomerated/non-aggregated 20 nm silica filler, non-agglomerated/non-aggregated 4 to 11 nm zirconia filler and aggregated zirconia/silica cluster filler (comprised of 20 nm silica and 4 to 11 nm zirconia particles).

### 2.1. Preparation of Direct Placement RBC Samples

Standardised discs (2 mm × 10 mm diameter) were fabricated from both direct-placement RBCs groups (Direct-COM and Direct-Control). These were polymerised with an LED photocuring calibrated unit (SmartLite FOCUS, Dentsply Sirona, York, PA, USA) in accordance with standards set by ISO 4049:2009 and manufacturer’s guidelines (40 s at 2 mm depth increments with overlapping curing zones). As per routine clinical placement, no attempt was made to prevent the formation of the oxygen inhibition layer, as this would be routinely removed during the finishing and polishing regimes (and released in the microparticle waste stream).

The discs were immediately ground using standard diamond dental burs (10–20 microns) in a water-cooled dental air turbine (SMARTtorque S619L, Kavo Dental, Bismarckring, Germany) to simulate clinical applications.

### 2.2. Preparation of CAD/CAM RBC Samples

Two CAD/CAM materials (CAD/CAM-COM and CAD/CAM-Control) were used in the form of ingots. The fabrication of Lava Ultimate (CAD/CAM-COM) is a manufacturer’s proprietary process involving silica particles of 20 nm diameter and zirconia particles of 4 to 11 nm diameter (approximately 80% by weight), treated with a silane coupling agent and incorporated within a unique resin matrix. Polymerisation of these blanks requires an industrial proprietary heat treatment process.

The control CAD/CAM RBC (CAD/CAM-Control) was fabricated using the same formulation as the direct-placement control material with silanated silica (approximately 75% by weight) designed for hybrid RBC applications (10–50 μm and 40 nm) (Table 1). CAD/CAM-Control RBC ingots were polymerised in the dental laboratory using a curing unit (Solidlite V, Shofu Corp, Osaka, Japan) for 5 cycles of 10 min to optimise the degree of monomer conversion. These ingots were not protected from developing an oxygen-inhibition layer and were subsequently lightly polished to remove any possible unpolymerised oxygen-inhibited surface, in a manner akin to the fabrication of commercial RBC CAD/CAM blanks.

All ingots were milled in a dry CAD/CAM unit (Roland DWX-50, Roland DG (U.K.) Ltd., Clevedon, UK). Sample cross-contamination was avoided by milling at separate times with deep-cleaning of the unit prior to each session.

### 2.3. Characterisation of RBC Samples

The particles from all samples, direct-placement and CAD/CAM RBCs, were collected, weighed and stored for either immediate characterisation or placed in solution (municipal tap water) for 12 months to simulate environmental release into water effluent systems.

The techniques utilised for the characterisation of RBC microparticles in wastewater samples were scanning electron microscopy (SEM), laser diffraction particle size analysis (PSA), micro-Fourier transform infrared (FTIR) spectroscopy and potentiometric titration.

### 2.4. Scanning Electron Microscopy (SEM)

Direct-COM RBC particles were mounted onto an aluminium pin-stub using a Leit-C sticky tab (Agar Scientific, Stansted, Essex, England), gold-coated using an Edwards S150B sputter coater (Edwards, Burgess Hill, West Sussex, England). SEM images were obtained from a Tescan Vega3 LMU Scanning Electron Microscope (Tescan-UK, Cambridge, Cambridgeshire, England) at an operating voltage of 10kv.

### 2.5. Particle Size Analysis

Particle size analysis of 1 g of freshly milled (not aged in solution) direct placement RBC samples was carried out using laser diffraction analysis in solution. Solutions of dispersed microparticles in deionised water were agitated in an ultrasonic bath for 120 s, 240 s and 360 s prior to laser diffraction analysis using a Malvern Mastersizer 2000 (Malvern Panalytical, Malvern, UK). Different agitation time frames were employed as forces of attraction rise rapidly below 10 μm which led to particle agglomeration, meaning the finer the sample the more difficult it is to separate the particles for analysis. The Brunauer–Emmett–Teller (BET) method was utilised to calculate the specific surface area (SSA) of the samples. A refractive index of 1.5 was selected as previous experimental composites have been shown to have refractive indices ranging from 1.47 to 1.53 [38].

### 2.6. Fourier Transform Infrared Spectroscopy (FTIR)

The particles from all direct placement and CAD/CAM RBC sampleswere washed with ultra-high quality (UHQ) water, and left to dry at room temperature for 8 h, prior to deposition on a stainless-steel flat sample holder for infrared micro-analysis. Reflectance micro-FTIR spectra were taken on a Perkin Elmer Spotlight 400 FT-IR Imaging System (Perkin Elmer, Waltham, MA, USA). Micro Fourier Transform Infrared (FTIR) spectra were collected over the 4000 cm^−1^ to 700 cm^−1^ wavenumber range, in reflectance mode, using a liquid nitrogen cooled Mercury–Cadmium–Telluride (MCT) array detector, at a resolution of 8 cm^−1^ and an aperture of 20 μm. Sixteen scans were taken for each pixel in the infrared maps, where each pixel corresponded to a square of 20 µm per side. A Kramer–Kronig transformation was not needed, as the resulting spectra did not present specular reflectance distortions.

Samples were also analysed on a Perkin Elmer Frontier Fourier Transform Infrared (FTIR) spectrometer (Perkin Elmer, Waltham, MA, USA), using a Perkin Elmer Attenuated Total Reflectance (ATR) accessory, consisting of a diamond crystal at a fixed angle of 45°. One-hundred spectra were collected over the 4000 cm^−1^ to 650 cm^−1^ wavenumber range, at a resolution of 4 cm^−1^.

### 2.7. Potentiometric Titrations

The RBC microparticle samples contain a variety of surface functional groups that could act as binding sites for protons. These functional groups can protonate or deprotonate when interacting with their immediate surroundings and as a result the particles develop a net pH-dependent charge [39,40,41,42,43,44]. Knowledge of these surface properties is crucial to understanding the interaction mechanisms between the microparticles and surrounding environmental species. The concentration and characteristics of proton active sites on the particle surfaces play an important role in this respect, as they are responsible for the surface binding ability [45]. All titrations of all samples were performed using a Metrohm Titrando 906 automatic titrator (Metrohm, Runcorn, UK) at 25 °C. The temperature was kept constant and continuously monitored during the titration. The titrator was set to add successive acid or base only after a drift equal or less than 5 mV min^−1^ was achieved; next, 0.05 g of each dry sample (washed four times with NaClO_4_) was suspended in a vessel with 25 mL CO_2_-free 0.1 M NaClO_4_ solution. The suspension was titrated with 0.1 M HCl to pH 3.5 and then with 0.1 M NaOH to pH 10.0. To test the reversibility of the protonation-deprotonation behaviour, the suspension was back-titrated with 0.1 M HCl from pH 10.0 to 3.5. The HCl and NaOH were previously standardised against primary standards. To calculate the acidity constant (pKa) values and the corresponding total concentration of the binding sites, data from two replicates of each titration curve were fitted using a program for determining surface protonation constants from titration data (Protofit 2.1 rev1) [46].

## 3. Results

### 3.1. SEM

**Figure 1:**
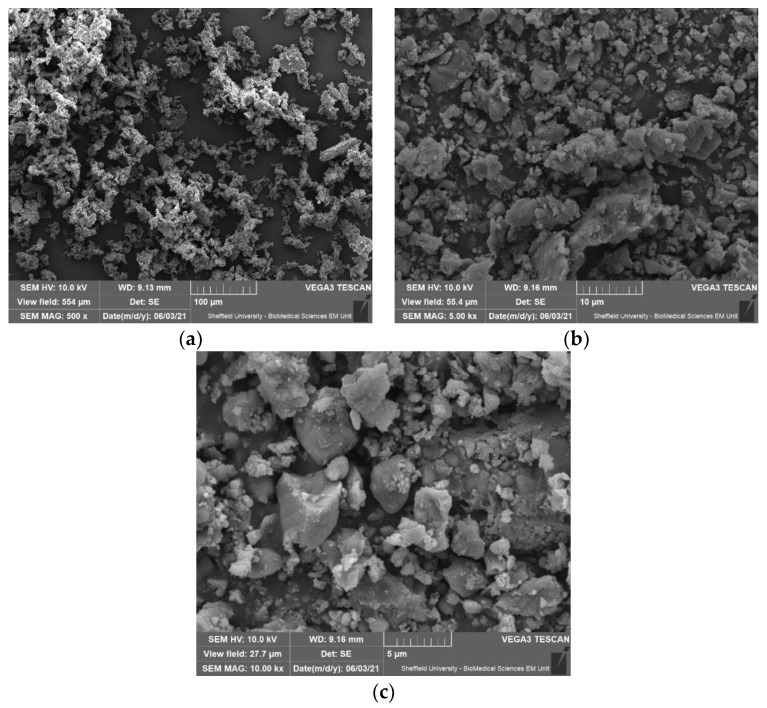
(**a**) (100 μm scale, upper left image). (**b**) (10 μm scale, upper right image). (**c**) (5 μm scale, lower image): SEM images of direct placement RBC (Direct-COM) ground using 10–20 micron diamond-coated burs in a water-cooled air turbine to simulate clinical applications.

The micrographs of the direct placement particulate sample are shown in Figure 1. The distribution and variation of the microparticles correlates with the data from the PSA results (Table 3). The images allow insight into the properties of the RBC particles to aggregate, however with clear indication of the presence of submicron-size particles which contribute to increasing the available reactive surface area.

### 3.2. Particle Size Analysis

Laser diffraction particle size analysis data of the Direct-COM and Direct-Control samples are detailed in Table 3. The particulate diameter measurements (Dx 10, Dx 50, Dx 90), span of particle distribution, surface area mean, volume mean diameter and specific surface area are the most relevant methods of analysing fine particulates such as RBC microparticles. Dx 50 is the size in microns that splits the distribution with half above and half below this diameter (median diameter). Similarly, Dx 90 is the size in microns where 90 percent of the distribution lies below this value, and 10 percent of the particulate sample lies below the value of Dx 10. The span of a volume-based size distribution is defined as Span = (Dx 90–Dx 10)/Dx 50 and gives an indication of how far the 10 percent and 90 percent points are apart, normalized with the midpoint. D [3,2] is the surface area mean (Sauter Mean Diameter) and is relevant to reactivity and elution of monomers from the smaller scale microparticles in the size distribution. D [4,3] is the volume mean diameter or volume moment mean (De Brouckere Mean Diameter) that reflects the size of the microparticles that constitute the bulk of the sample volume and is more sensitive to presence of larger particulates in the size distribution. Incremental increased agitation from 120 s to 240 s to 360 s resulted in corresponding increased specific surface area of all samples. Dx 50, Dx 90, Span, D [3,2] and D [4,3] are all less for Direct-COM compared to the Direct-Control material, presumably due to inclusion of nanoscale particulates. The maximum specific surface area of Direct-COM was seen after 360 s of agitation and was 1290 m^2^/kg compared with 1017 m^2^/kg for the control material. The median diameter of Direct-COM was 6.39 μm at 360 s agitation and 9.55 μm for the Direct-Control material; both being significantly smaller than the 5 mm threshold that defines microplastic pollutants and at the lower scale of the range of what defines a microparticle (1 to 1000 μm).

### 3.3. FTIR

Fourier transform infrared (FTIR) spectroscopy is one of the most suitable, reliable and commonly used methods for the characterisation, identification and quantification of microplastics in environmental samples [47,48]. FTIR microspectroscopy (micro-FTIR) is a tool that combines FTIR spectroscopy with microscopy, allowing the identification and comparison of infrared bands in smaller samples due to the improvement in spatial resolution. Reflectance micro-FTIR images are obtained by illuminating the surfaces of the material and collecting sufficient scattered radiation. This approach is particularly useful as it requires little sample preparation and enables the rapid analysis of thick and opaque samples.

**Figure 2:**
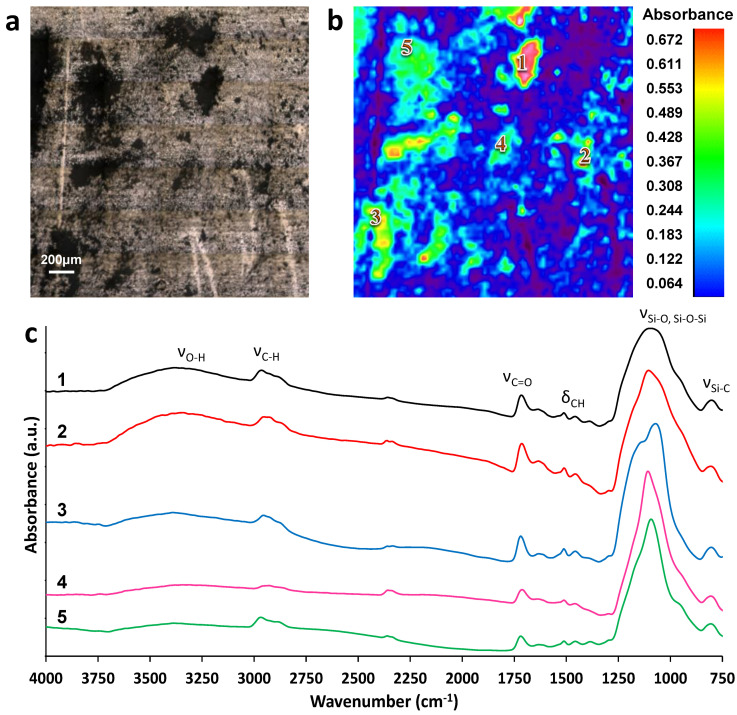
Reflectance micro-FTIR spectroscopy of Direct-COM microparticles on stainless steel surface: (**a**) Optical image of sample. (**b**) False-colour image showing the location of molecules with absorption bands between 4000 and 750 cm^−1^. Relative absorbance scale (a.u.) is shown on the right. (**c**) Reflectance micro-FTIR spectra of the area containing Direct-COM microparticles on a steel surface, at different points on the false-colour image. Spectra have been offset for comparative purposes.

Figure 2 shows an example of the image of the dried direct RBC Direct-COM microparticles on a stainless-steel sample holder, taken with the optical microscope, alongside the false-colour image of the same area, analysed using reflectance micro-FTIR spectroscopy.

Figure 2c shows the FTIR spectra at six different points in the scanned area between 4000 and 750 cm^−1^. Apart from the regions corresponding to the bare steel surface, all the observed particles presented infrared absorption bands, and the similarity between their spectra suggested that the scanned particles have similar composition.

The bands observed in the region between 3050 and 2700 cm^−1^ can be assigned to C–H stretching vibrations (υ_C–H_), corresponding to CH_3_ and >CH_2_ functional groups [49,50]. The O–H stretching signal (υ_O–H_) corresponding to the presence of hydroxyl groups can be assigned to a broad band around 3400 cm^−1^. Complementary information to support the presence of the C-H peaks can be found on the region between 1470 and 1300 cm^−1^, where bending vibrations of C–H, >CH_2_ and –CH_3_ groups are observed. The bands around 1730 cm^−1^ are assigned to the stretching C=O (υ_C=O_) of carbonyl groups [51,52]. The strong bands around 1045 and 900 cm^−1^ have been usually attributed to stretching Si–O and Si–O–Si. Furthermore, bands around 790 cm^−1^ are commonly assigned to Si–C vibrations [53,54].

**Figure 3:**
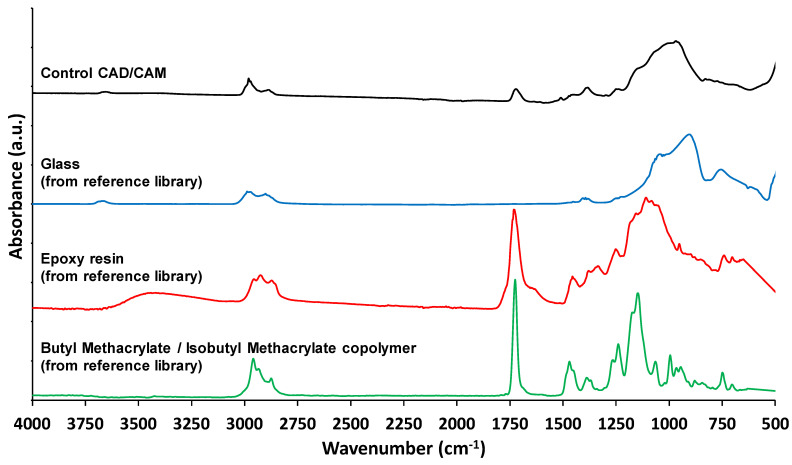
ATR-FTIR spectra of unaged, freshly milled CAD/CAM-Control and “Glass”, “Epoxy resin” and “Butyl Methacrylate/Isobutyl Methacrylate copolymer”, from the reference library.

The penetration depth of the infrared beam on reflectance micro-FTIR depends on the scanned material, but usually ranges from 2 to 5 µm. The penetration depth of the infrared beam on ATR-FTIR measurements depends on the sample, wavenumber, internal reflection element used and angle of incidence, and are normally in the range of around 300 nm at 1000 cm^−1^ [55]. Therefore, ATR-FTIR is a more surface-sensitive technique than reflectance micro-FTIR. From these data, comparison to spectra held on the Fluka spectral database within the Perkin Elmer Spectrum software were made (Figure 3). After comparison with ATR-FTIR spectra of similar materials held on the Fluka database, it is evident that these microparticles are sufficiently unique to be identified, where the spectra of glass, epoxy resin and butyl methacrylate are compared to the freshly milled CAD/CAM-Control RBC.

**Figure 4:**
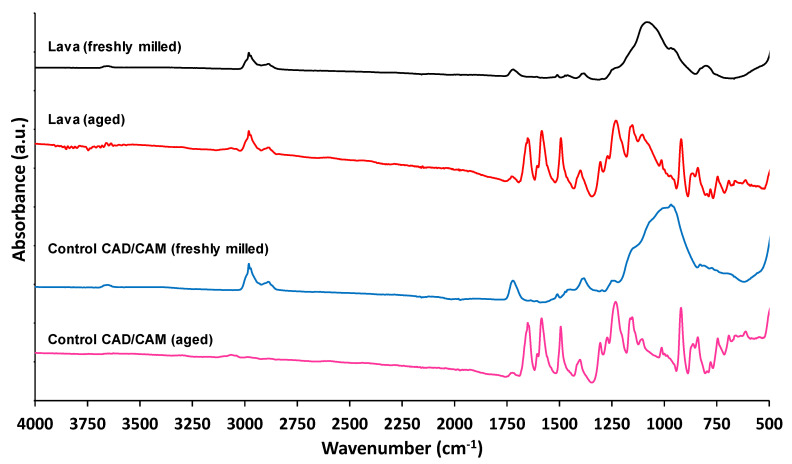
ATR-FTIR spectra of the CAD/CAM-COM and CAD/CAM-Control freshly milled and aged samples, for better comparison.

Comparison of freshly milled CAD/CAM particulates (COM and Control) versus those aged in microcosms containing water for 12 months is shown in Figure 4. There is consistency between the spectra in comparison of the COM and control materials and also the impact of ageing. The ATR-FTIR spectra of the aged CAD/CAM-COM and CAD/CAM-Control microparticles suggest that the outermost surfaces of the particles have been altered. Theories as to what this has been caused by include hydrolysis and leaching of surface monomers, adsorption of organic compounds such as eluted monomers, or the establishment of a layer of proteinaceous biofilm [56,57].

Infrared absorption bands that could support the presence of biofilm in the surface of the aged samples (Figure 4) include the amide I and II bands at 1650 and 1570 cm^−1^, respectively, the first one due to stretching C=O (ν_C=O_) of amides associated with proteins and the latter a combination of bending N-H (δ_N–H_) of amides and also contributions from stretching C=N (ν_C=N_) groups [58]. The absorption at 1250 cm^−1^ in the aged samples could be attributed to the double bond stretching of >P=O of general phosphoryl groups, phosphorylated proteins, polyphosphate products, and phosphodiester of nucleic acids [40,59]. The region between 1200 and 800 cm^−1^ is usually dominated by the complex superposition of vibrations corresponding to the C–O–C and C–O–P stretching, where specific band assignments are very difficult. The presence of carbonyl, amide and phosphoryl functional groups also contribute to the acid–base exchange reactivity observed in the potentiometric titration experiments [40,43,60] (see next section).

### 3.4. Potentiometric Titrations

**Figure 5:**
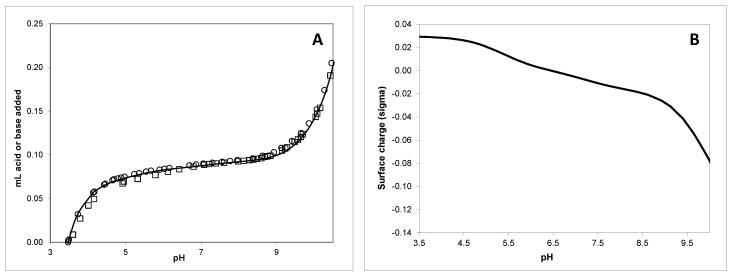
Potentiometric titration data for particle suspensions for aged CAD/CAM-COM in NaClO_4_ solution. (**A**) Titration data and model fitting using a Non-Electrostatic Model. Circle symbols correspond to the forward titration data and square symbols correspond to back titration. (**B**) Surface charge, as a function of pH.

Examples of the potentiometric titration curves are presented in Figure 5A,B.

The concentration of deprotonated sites is standardised per mass of sample (mol g^-1^) and calculated according to Fein et al. [60] as follows:

[H+]_consumed/released_ = (C_a_ − C_b_ − [H^+^] + [OH^−^])/m_b_ (1)

where m_b_ is the concentration of particles in the suspension (g L^−1^); C_a_ and C_b_ are the concentrations of acid and base added at each step of a titration, respectively; and [H^+^] and [OH^−^] represent molar species concentrations of H^+^ or OH^−^. In order to calculate the acidity constants and the total concentration of each binding site, data from the titration curves were fitted using the software ProtoFit 2.1 using a Non-Electrostatic Model (NEM).

The titrated suspensions exhibited a protonation–deprotonation behaviour over the whole pH range studied (see Figure 5A,B). In this figure, the 12-month-aged CAD/CAM-COM particle data are shown as a representative illustrative example. No evidence of saturation was found with respect to proton adsorption, indicating that, even at pH 3.5, full protonation of the functional groups on the surface was not achieved. The shape of the titration curves obtained suggested the presence of functional groups with close acid-base pKa values [40,61], showing that although some small variability could be perceived in each set of the same sample, essentially reproducible results were obtained (the variation between the titration curves was below 6% of [H+]_exchanged_ between pH 3.5 and 10). Although a small hysteresis could be observed between acid and base titrations at the same ionic strength, results from reverse titrations did not vary strongly and suggested a reversible proton adsorption/desorption reaction. Table 4 summarises the pKa values and surface site densities for the different tested samples. 

The obtained pKa values are representative of silica/silanol groups for pK_1_, hydroxyl groups for pK_2_ and carboxylic groups for pK_3_ [61]. The existence of pH of zero proton charge (pH_zpc_) indicated that all samples of the particles developed a positive net charge at low pH values [62]. The pH_zpc_ ≈ 5–6 also indicated that the particles are negatively charged at neutral pH7 and electrostatic attraction with positive-charged surfaces, ionic species or metals may be favourable.

The preliminary results of potentiometric titration experiments on the studied samples indicated that the surface groups capable for potential surface binding are sites involving carboxyl groups (p*K* ≈ 3–5), sites involving silanol/silica groups *(*p*K* ≈ 6–7) and sites involving hydroxyl or amine groups (p*K* > 8). These findings are in agreement with previous studies on colloidal surfaces [44,45,46].

## 4. Discussion

This study aimed to characterise various examples of dental RBC microparticulate waste following release into the environment by means of PSA, SEM imaging, reflectance micro-FTIR, ATR-FTIR and potentiometric titrations. RBC microparticulate waste is a small contribution to environmental pollution when compared to plastic microparticle waste from other industries. Moreover, this research is pertinent as the healthcare sector has a responsibility to be sustainable with consideration of potential harm and more importantly, avoidance of harm.

Previous reported research in this field has primarily focused on the production of RBC dust from dental office/laboratory grinding procedures. A recent systematic review of particulate production and composite dust during routine dental procedures identified only five articles that contributed to the meta-analysis [63]. These papers did not consider environmental release, with a predominant focus of the reviewed literature on dust characterisation, outcome of potential inhalation and cytotoxicity, and the methods used to generate the RBC dust. Only one of these papers in this review used ATR-FTIR and was concerned with orthodontic adhesive particles [64] justifying the need for further research to increase our understanding of RBC particulates and environmental release pathways.

From the results obtained in this study, there are a series of specific findings and implications. The PSA data (Table 3) revealed that the particulate material generated for the direct RBC commercial and control material was in the microplastic size range of <5 mm (0.5 μm to 1000 μm) with median particle sizes ranging from 6.39 to 10 μm for all samples. Utilisation of nano-scale particulates in the commercial RBC (with 5–100 nm filler particles and classified as “nano-fill” [65]) created smaller microparticles, however aggregation in solution resulted in most particles observed as micro-scale. The SEM images confirmed this, showing aggregation of the Direct-COM RBC particles in addition to the presence of submicron particles. This current study sought to replicate clinical conditions with the use of water spray during the grinding process of the RBC samples. This is akin to common clinical applications that generate greater volumes of microparticulate waste, such as adjusting/polishing/removing restorations or preparing teeth for extra coronal prostheses. It is recognised that grinding with water spray significantly decreases RBC nanoparticle release but does not eliminate it [66]. In addition, it has been previously proposed that the use of water coolant when grinding RBC theoretically contributes to the formation of larger particle sizes [67]. The potentiometric titrations data from this study demonstrated generation of surface charge, and there is an expectation from these data that aggregation or clumping of particles occurs due to electrostatic attraction.

The PSA data highlight the commercial direct-placement RBC had the smallest particle size and greatest specific surface area across all agitation times compared to the control, with particles in the nano-scale raising concerns highlighted by other studies investigating similar RBC materials [67,68]. A proportion of the particles detected in the higher size range were due to aggregation of particles. The large surface area of the particles identified in the data highlights a resultant accelerated elution of monomer into the environment occurs. The significance of this is clear, especially with materials containing BisGMA (which is associated with the release of BPA [7]) as BPA elution over a prolonged period of time has been previously detected in samples of water (aged >6 months) containing RBC particulates [69].

The effect of time in solution on the microparticles (representing environmental release) is evident through the comparison of the ATR-FTIR spectra for the commercial and control CAD/CAM materials (Figure 4). The experimental ageing process used to simulate release into the environment had the effect of altering the surface chemistry of RBC microparticle surfaces consistently for all samples tested. A plausible explanation for this difference is due to either microbial biofilm colonisation of the particulates, the elution of monomers or a combination of the two. It has been previously demonstrated that increased amounts of monomers are released from RBCs in untreated landfill leachate when compared to abiotic landfill leachate, demonstrating the impact of bacterial interaction with RBC [70]. Elution of monomers from direct placement and CAD/CAM RBC is well represented in the literature but has focused mainly on macro-scale samples such as disc-shaped or blocks of specimens [71,72,73]. Few studies have investigated the elution of monomers from microparticulate RBC, and until recently, even fewer have considered an environmental impact of this phenomenon [72].

The data from the potentiometric titrations of the RBC microparticulate samples indicated that although RBC is a heterogenous material made up of glass in a resin matrix, the uniformity of manufacturing and the commonly utilised chemical compounds that make up the majority of these materials reveal minimal variance between the CAD/CAM commercial or control samples. Aged materials showed a capability of protonation–deprotonation behaviour, and it is therefore possible to potentially electrostatically attract these particles in charged filters after long-term release in the environment (Figure 5).

Having established the potential pollutant impact and release pathways for microparticles arising from RBC, it is pertinent to consider appropriate management strategies. A logical first-step approach to reducing the environmental burden of RBC particulate waste is to reduce the number of restorations requiring placement and subsequent replacement. Such a strategy should focus on a strong preventive ethos that obviates the need for restoring teeth in the first instance combined with high quality operative care and effective maintenance protocols that seek to maximise durability. Beyond this, attention should focus on recovery of waste at the point of production with consideration to recycling capabilities. Particles generated from dry-milled CAD/CAM applications should be disposed of responsibly and not into municipal waste, which would subsequently be placed in landfill. Collection and processing systems should be put in place for this particulate waste. For water-based microparticulate waste, there is clear scope to consider filtration mechanisms. Such systems have been shown to optimally remove BPA from water samples using activated carbon such as that used in a recent study by Polydorou et al [74]. The surface of the catalytic carbon filter in this study was highly positively charged. As noted previously, the potentiometric titrations carried out in our study highlighted that at neutral pH RBC particulates are negatively charged, explaining catalytic carbon’s good adsorptive properties. A further development would be to consider how intentional variance in the pH of the wastewater from dental surgeries would improve filtration efficiency. Given the potential impact of BPA as an important hazardous substance released from RBC due to its estrogenic impacts even in small dosages [75], there is a clear research need to investigate its access to food webs and potential impacts.

Polydorou et al.’s study complements our current work described in this article, highlighting the recognised impact of the large surface area of waste RBC particulates and elution of constituent monomers and BPA, in addition to suggesting a method for recovery filtration to prevent contamination of effluent water systems.

The characterisation of microparticles arising from RBCs adds to our baseline knowledge in this field and prompts further research recommendations: (i) The need for a comprehensive analysis that characterises and quantifies the environmental impact of the eluted monomers and BPA from all RBC sources with due consideration to the multiple pollution pathways. (ii) The micro-FTIR and ATR-FTIR data for all samples and comparison to similar materials held on the Fluka database (PerkinElmer Spectrum software) showed that the RBC samples were unique enough to be identified. This provides an opportunity to detect RBC microparticulate waste downstream of dental practices and laboratories. Identification and quantification of microplastics in wastewater using micro-FT-IR imaging has been accomplished, and this can now be applied to dental RBC microparticles [76]. (iii) There is a clear need to pursue the development of more environmentally sustainable dental materials and improved management of associated microparticulate waste.

## 5. Conclusions

In this study, we have characterised microparticulate waste arising from grinding direct placement and CAD/CAM resin-based composites. RBC microparticles are distinct enough to be identified using FTIR, allowing potential testing of wastewater from dental surgeries. It is also clear that particulate waste is altered when released into the environment due to anticipated elution of constituent monomers, potentially exacerbated by biofilm, bacterial interaction and surface area. There is potential for RBC microparticles to become toxic polluters and/or vectors for pollution due to the reactivity highlighted in this work. Remediation strategies should focus on reduced use of materials and effective recovery with potential recycling.

The ability to manipulate the charged nature of these particles provides an opportunity for the recovery of these pollutant particles at point of generation. This would specifically be within dental surgeries where the greatest source of particulate plastic waste from an RBC source originates from, the removal or replacement of failed RBC restorations.

## Figures and Tables

**Table 1:** Description of samples tested.

Samples	Clinical Use	Nomenclature	Description
Control	Direct-Placement RBC	Direct-Control	See Table 2
	Machined CAD/CAM RBC	CAD/CAM-Control	See Table 2
Commercial	Direct-Placement RBC	Direct-COM	Filtek Supreme XTE, shade A3 (3M Oral Care)
	Machined CAD/CAM RBC	CAD/CAM-COM	Lava Ultimate, size 14L shade A3 (3M Oral Care)

**Table 2:** Constituents of Control RBC (Direct placement and CAD/CAM; with a resin/filler component ratio 3:1.

RBC Component	Role	Percentage
BisGMA	Monomer	8.75
UDMA	Monomer	8.75
TEGDMA	Monomer	7.3
Camphorquinone	Photoinitiator	0.075
4-dimethylaminobenzoic acid ethyl ester (DMABE)	Accelerator	0.075
3,5-di-tert-butyl-4-hydroxytoluene (BHT)	Inhibitor	0.0003
2-hydroxy-4-methoxybenzophenone (HMBP)	Photostabiliser	0.00015
Silane treated silica (10–50 μm and 40 nm)	Filler	75

**Table 3:** Particle size analysis of direct-placement RBCs (Direct-COM and Direct-Control).

RBC Particulate Sample	Dx 10	Dx 50 (Median Diameter)	Dx 90	Span (Dx 90–Dx 10/Dx50)	D [3,2] (Surface Area Mean)	D [4,3] (Volume Mean Diameter)	Specific Surface Area (BET Method)
Direct-COM 120s US agitation	2.37 μm	7.13 μm	27.4 μm	3.509	5.24 μm	12.7 μm	1145 m^2^/kg
Direct-COM 240s US agitation	2.22 μm	6.65 μm	23.8 μm	3.247	4.86 μm	10.8 μm	1234 m^2^/kg
Direct-COM 360s US agitation	2.13 μm	6.39 μm	22.6 μm	3.208	4.65 μm	10.4 μm	1290 m^2^/kg
Direct-Control 120s US agitation	2.84 μm	10.0 μm	47.6 μm	4.472	6.57 μm	22.9 μm	912.9 m^2^/kg
Direct-Control 240s US agitation	2.58 μm	9.52 μm	39.9 μm	3.918	6.03 μm	16.5 μm	994.7 m^2^/kg
Direct-Control 360s US agitation	2.49 μm	9.55 μm	40.2 μm	3.945	5.90 μm	37.1 μm	1017 m^2^/kg

**Table 4:** Comparison of deprotonation constants and surface site concentrations for the titrated samples (^a^ NEM = non-electrostatic model).

Sample	Ageing	pK_1_	pK_2_	pK_3_	C_1_ (× 10^−4^ mol/g)	C_2_ (× 10^−4^ mol/g)	C_3_ (× 10^−4^ mol/g)	Model ^a^	pH_zpc_
Direct-COM	Fresh	6.54 ± 0.63	9.72 ± 0.20	4.48 ± 0.31	0.056 ± 0.03	0.363 ± 0.13	0.157 ± 0.19	NEM	5.62 ± 0.74
Direct-COM	Aged	7.04 ± 0.19	10.08 ± 0.16	4.46 ± 0.90	0.058 ± 0.02	0.320 ± 0.03	0.061 ± 0.05	NEM	5.72 ± 0.23
CAD/CAM-Control	Aged	6.95 ± 0.41	10.08 ± 0.12	4.44 ± 1.32	0.029 ± 0.02	0.162 ± 0.09	0.077 ± 0.07	NEM	5.88 ± 0.79
CAD/CAM-COM	Fresh	6.21 ± 0.42	9.70 ± 0.48	3.83 ± 0.49	0.035 ± 0.02	0.165 ± 0.02	0.063 ± 0.01	NEM	5.17 ± 0.37
Direct-Control	Fresh	7.65 ± 1.85	9.90 ± 0.36	5.64 ± 1.09	0.083 ± 0.02	0.241 ± 0.03	0.158 ± 0.11	NEM	6.74 ± 1.56
Direct-Control	Aged	7.41 ± 0.78	9.78 ± 0.43	4.79 ± 0.28	0.046 ± 0.03	0.292 ± 0.20	0.133 ± 0.06	NEM	6.35 ± 0.20

## Data Availability

The data presented in this study are available on request from the corresponding author.
